# Mechanism underlying linezolid-induced peripheral neuropathy in multidrug-resistant tuberculosis

**DOI:** 10.3389/fphar.2022.946058

**Published:** 2022-09-09

**Authors:** Yuan Yuan, Jinmeng Li, Yanhong Chen, Qingshan Cai, Yingying Xu, Luting Lin, Yazhen Lang, Suhang Guo, Ruoying Zhang, Xinjun Cai

**Affiliations:** ^1^ Zhejiang University School of Medicine, Affiliated Hangzhou Chest Hospital, Hangzhou, Zhejiang, China; ^2^ Laboratory Animal Center of Zhejiang University, Hangzhou, Zhejiang, China; ^3^ College of Pharmacy, Zhejiang Chinese Medical University, Hangzhou, Zhejiang, China

**Keywords:** linezolid, multidrug-resistant tuberculosis, peripheral neuropathy, schwann cells, autophagy

## Abstract

Multidrug-resistant tuberculosis (MDR-TB) remains a main global health concern as there is no comprehensive therapeutic intervention yet and numerous adverse effects follow the therapeutic process. In recent years, linezolid has been frequently used for treating MDR-TB. However, peripheral neuropathy associated with linezolid has reduced patient compliance. The current study explored the mechanism underlying linezolid-induced peripheral neuropathy in MDR-TB. Autophagy plays a neuroprotective role against peripheral nerve injury. We hypothesized that autophagy might also play a neuroprotective role against linezolid-induced peripheral neuropathy. In this study, we collected 12 questionnaires from MDR-TB patients in our hospital, and 10 of them developed linezolid-induced pain. The pain is mainly concentrated in the feet and accompanied by numbness. Subsequently, we used Sprague-Dawley (SD) rats and Schwann cells (SCs) to explore the mechanism. We found that linezolid causes a sparse arrangement of sciatic nerve tissue with associated loss of neurons, myelin sheaths, and down-regulation of LC3B expression. These results were also confirmed by *in vitro* experiments, showing that linezolid inhibited the proliferation of SCs. And the expression of P-AKT and P62 was elevated, and the expression of LC3B declined compared with the control group. Moreover, chloroquine (CQ), an autophagy inhibitor, also exhibited experimental results similar to linezolid. In summary, we conclude that linezolid-induced peripheral neuropathy is associated with the inhibition of autophagy flux.

## Introduction

Multidrug-resistant tuberculosis (MDR-TB) remains considerably challenging to treat. Notably, China has the second highest TB burden globally (WHO, 2021). In 2019, nearly half a million people worldwide developed rifampicin-resistant tuberculosis (RR-TB), and 78% of these had MDR-TB (WHO, 2019). Although MDR-TB is curable, only 54% of patients recover entirely (Bolhuis et al., 2018). Therapeutic options for MDR-TB have been limited due to insensitivity to rifampicin and isoniazid. Currently, clinical treatment requires at least 18 months of second-line drug courses. Moreover, these drugs can lead to numerous adverse reactions, which reduce patient compliance.

Linezolid has become the mainstay of oxazolidinone antibiotic therapy due to its favorable properties, such as high oral bioavailability, good tissue penetration, and minimal drug resistance (Diekema & Jones, 2001; Lee et al., 2019; O'Daniel et al., 2014). It has been used to treat Gram-positive bacterial infections. In recent years, linezolid has been frequently used for the treatment of drug-resistant strains of *Mycobacterium tuberculosis*. The World Health Organization lists linezolid as the recommended drug for more extended MDR-TB regimens. This suggests that patients with MDR-TB require, in theory, 18–20 months of linezolid administration (Mirzayev et al., 2021).

However, some patients discontinue linezolid early due to intolerance (Berry, Yates, Seddon, Phillips, & du Cros, 2016; Conradie et al., 2020). Short-term treatment with linezolid has an overall favorable safety profile, as indicated by clinical trials and post-marketing surveillance (Rubinstein et al., 2003; Lee et al., 2019). On the contrary, the use of linezolid for >28 days resulted in more severe adverse events, such as peripheral neuropathy, myelosuppression, or optic neuritis (Frippiat, Bergiers, Michel, Dujardin, & Derue, 2004; Zhang et al., 2015; Garrabou et al., 2017). According to a meta-analysis, 64% of patients with MDR-TB discontinued linezolid permanently due to peripheral neuropathy (Lan et al., 2020). Therefore, the development of peripheral neuropathy has limited the total duration and therapeutic dosage of linezolid, resulting in treatment failure. Reports published recently have proposed that linezolid is strongly correlated with autophagy (Abad et al., 2019; Tasneen et al., 2021). Nevertheless, the association between linezolid-induced peripheral neuropathy and autophagy remains undetermined. To address this clinical issue, we used SD rats and SCs to explore the potential neurotoxic mechanism of linezolid.

## Materials and methods

### Clinical research ethics and informed consent

The ethics committee has approved our study protocol for collecting patient clinical information from Affiliated Hangzhou Chest Hospital, Zhejiang University School of Medicine. This protocol was strictly followed throughout the entire experimental process. Informed consent has been obtained from the patients.

### Clinical records and information collection

Overall, 12 eligible patients with TB were recruited from Affiliated Hangzhou Chest Hospital, Zhejiang University School of Medicine, between September 2021 and December 2021. The diagnostic criteria for linezolid-induced peripheral neuropathy were as follows: a history of MDR-TB with typical TB symptoms; imaging and laboratory evidence; and linezolid administration for >3 months at a daily dose of ≥600 mg. The exclusion criteria were as follows: 1) HIV positivity; 2) presence of severe heart, liver, kidney, and other organ diseases contraindicating anti-TB drug administration; 3) age <14 years; 4) non-TB branch *Bacillus* infection; and 5) loss to follow-up after treatment. Critical clinical information on pain type, duration, and pain characteristics of linezolid-induced peripheral neuropathy was collected from patients using questionnaires.

### Experimental animals and drug administration

All animal experimental procedures were approved by the Laboratory Animal Ethics Committee of Zhejiang University. Adult male SD rats (200–250 g) were obtained from the Laboratory Animal Center of Zhejiang University. Five rats per cage were housed under the following controlled environmental conditions: 23°C ± 2°C temperature, 35%–60% humidity, and a 12:12 h light: dark cycle. The animals had free access to water and food. After 1 week of acclimatization, all the experimental animals were used for animal experiments. The rats were then randomly divided into four groups: Sham group (*n* = 4), 250 mg/kg-linezolid treated group (*n* = 5), 125 mg/kg-linezolid treated group (*n* = 5), 50 mg/kg-linezolid treated group (*n* = 5). The body weight of the rats was measured weekly. Four weeks later, the rats were performed behavioral tests. Afterward, the rats were anesthetized with pentobarbital sodium (50 mg/kg) and sacrificed. For drug administration, linezolid (Pfizer, New York, United States) was dissolved in saline, and the rats were treated through intragastric administration at the dose of 250, 125, and 50 mg/kg/d for 4 weeks. These doses in rats have been described previously (De Vriese et al., 2006).

### Basso beattie and bresnahan scores

The Basso Beattie and Bresnahan (BBB) scale was adopted to evaluate the locomotor ability of the hind limbs in spinal cord injury models and is also applicable to peripheral nerve injury models (Pertici et al., 2014; Watzlawick et al., 2014; Li et al., 2018). The scores were divided into 22 levels of bilateral hind limb motor function. A score of 0 indicates total paralysis, whereas a score of 21 indicates normal walking. A double-blind method was conducted to experiment. The main observation parameters included a range of motion, number of joint movements, degree of weight-bearing, body balance, and front and hind limb coordination. After administration with linezolid for 4 weeks, the rats were placed in an open and quiet field and allowed to explore autonomously. After the exploration activity was over, each rat was independently observed for 5 min. At the same time, the motor function of both hind limbs was assessed, and the observation scores were recorded.

### Walking track analysis

Four weeks after linezolid administration, the rats were performed walking track analysis (Amado et al., 2008; Tang et al., 2013). The rats’ hind limbs were dipped in dark ink and allowed to walk independently through a black tunnel (100 cm * 10 cm). White paper at the bottom of the tunnel recorded the behavior of the rats’ hind limbs. The same investigator who never participated in the behavioral experiments repeated the experimental process thrice.

### Immunofluorescence staining

Sciatic nerves were immersed in 4% paraformaldehyde (PFA; Gibco, California, United States) for 24–48 h, after which the sciatic nerves were dehydrated with ethanol gradient and embedded in paraffin. 5 μm sciatic nerve sections were cut from paraffin and rehydrated. Then the sections were incubated in 3% H_2_O_2_ for 15 min and blocked by 5% bovine serum albumin (BAS; Beyotime, Shanghai, China) for 30 min. Subsequently, the sections were incubated with primary antibody at 4°C overnight. After washing with phosphate-buffered saline (PBS; Gibco, California, United States) with 0.1% Tween-20 (Aladdin, Shanghai, China), the sections were incubated with Alexa Fluor 647 (1:1000, Abcam) or Alexa Fluor 488 (1:1000, Abcam) as secondary antibodies at 37°C for 60 min. Cellular nuclei were stained with 4ʹ,6-diamidino-2-phenylindole (DAPI; Yesen, Shanghai, China). All fluorescence images were obtained under the Nikon ECLIPSE 80i (Nikon, Tokyo, Japan). The following primary antibody were used: MBP (1:1000, CST), NF-200 (1:1000, CST), LC3B (1:1000, CST).

### Cell culture and treatment

RSC 96 (ScienCell Research Laboratories, Shanghai, China), which is the rat SCs line, was cultured in Dulbecco’s Modified Eagle Medium (DMEM; Gibco, California, United States) containing 1% penicillin/streptomycin solution (Gibco, California, United States) and 10% fetal bovine serum (FBS; Gibco, California, United States) in a humidified incubator (37°C, 5% CO_2_). In the confirmation phase of linezolid, experiments were divided into four groups: control group, 250, 125, and 62.5 μg/ml. Schwan cells were inoculated in 96-well plates (3× 10^3^ cells/well) and 6-well plates (1× 10^3^ cells/well) for cell viability analysis and colony-forming cell assays, respectively. Subsequently, SCs were seeded into 6-well plates at a density of 1 × 10^5^ cells/well to explore the mechanism. The selected wells were given CQ (40 μM; Selleck Chemicals, Houston, Texas, United States) and linezolid (250 μg/ml) for 72 h. Otherwise, SCs were treated with CQ 2 h before linezolid treatment.

### Cell counting kit-8 assay

Cell viability assay was evaluated with cell counting kit-8 (CCK-8; MCE, Monmouth Junction, NJ, United States). Discarded the blank medium and drug-containing medium in the 96-well plate, washed it three times with PBS, and added 100 μl of fresh medium. After that, 10 μl of CCK-8 solution was added to each well. Placed in the incubator for a proper time. When the color changed to orange, a microplate reader measured the absorbance values via spectrophotometry at 450 nm. At least three replicate wells were set for each group of cells.

### Colony-forming cell assays

SCs in each group at the logarithmic growth stage were collected to prepare suspension for cell counting and dilution. The cells were then inoculated in a 6-well plate and gently rotated so that the cells were evenly dispersed. After cell adhesion, different concentrations of linezolid solutions were added, while the control group was added PBS. Incubate in an incubator for 14 days, and replace fresh medium every 3 days. The culture was terminated after 2 weeks, and the medium was abandoned. The cells were fixed with methanol for 15 min and stained with Giemsa dye for 10 min. After washing away the excess dye, the cells were counted, and the number of clones formed was observed under a light microscope.

### Western blotting analysis

Proteins were extracted from RSC96 cells using a lysis buffer containing protease and phosphatase inhibitor cocktail (10 μl/ml, Beyotime, Shanghai, China). After centrifugation for 10 min at 12,000 rpm, the supernatant was taken, and the protein concentration was detected by the Bradford kit (Abcam, Cambridgeshire, England). Protein lysates containing 40 μg of protein were separated on 10% or 12% SDS–polyacrylamide gels and transferred to polyvinylidene fluoride membranes (Merck Millipore, Darmstadt, Germany). After blocking with 5% skim milk for 90 min at room temperature, the membranes were incubated with primary antibody at 4°C overnight. Three times washing with TBST (TBS with 0.05% tween 20), the membranes were incubated with the corresponding secondary antibody. The primary antibodies used in this experiment were as follows: p-AKT (1:1000, CST), p62 (1:1000, CST), LC3A/B (1:1000, CST), and GAPDH (1:1000, CST).

### Statistical analyses

All data obtained in this study were the average of at least three repetitions and were plotted as means ± SEM. Comparisons between two groups were performed using a one-way analysis of variance followed by Turkey’s multiple comparison test, with *p* < 0.5 indicating statistical significance.

## Results

### Clinical information collection

**FIGURE 1:**
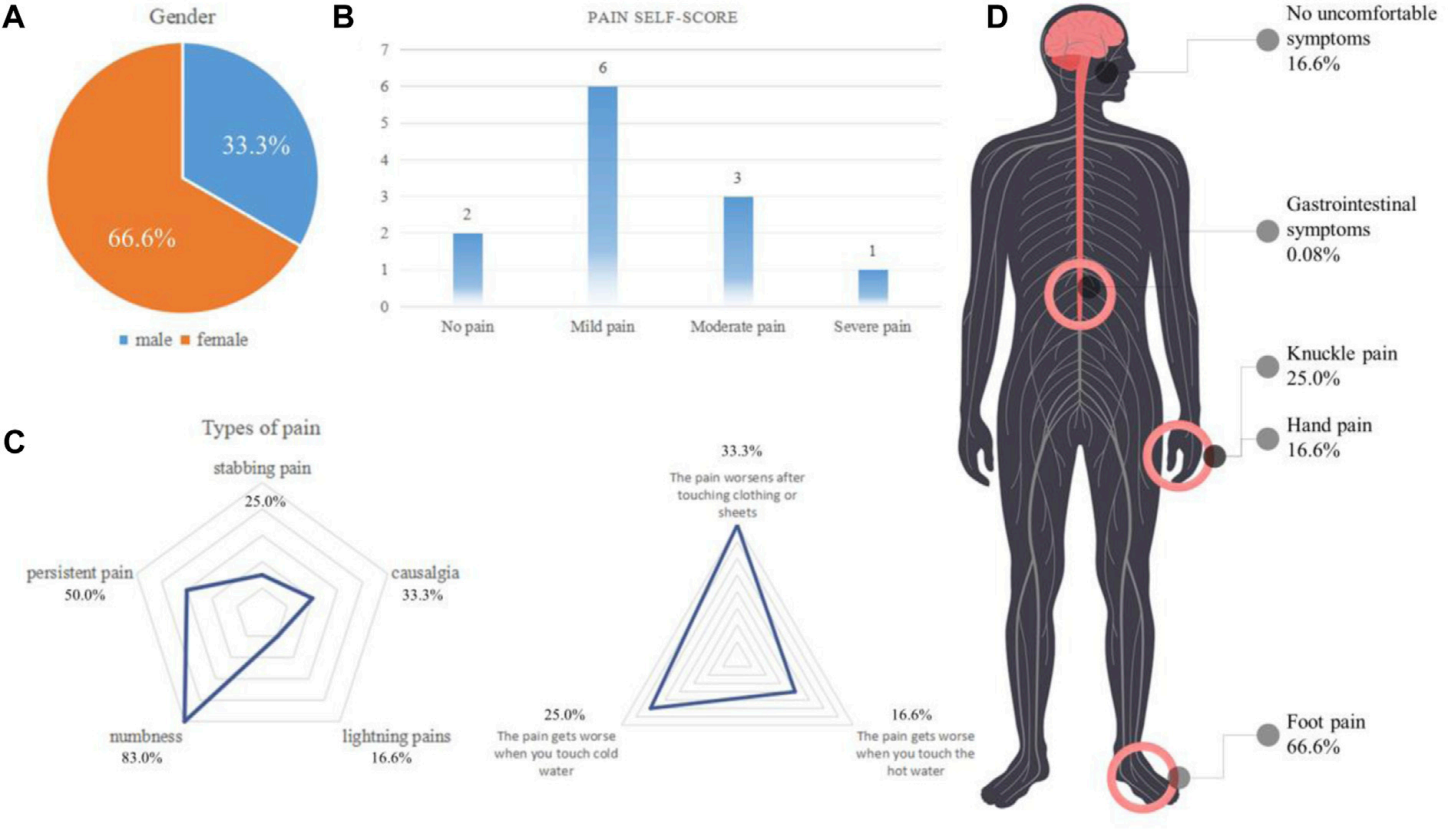
Analysis of the types and characteristics of linezolid-induced pain (*n* = 12). **(A)** Sex ratio of the included patients. **(B)** Patient pain levels. **(C)** Analysis and proportion of pain types. **(D)** Location and proportion of pain.

From September 2021 to December 2021, we collected a total of 12 valid questionnaires. The average age of the patients was 34 years. The ratio of men to women respondents was 1:2 (Figure 1A). Through the questionnaires, we determined that 10 patients developed linezolid-induced pain, which mainly manifested as mild pain (Figure 1B). In addition to the sensation of pain, 83.0% of patients developed numbness (Figure 1C). Besides, 66.6% of patients reported that linezolid-induced pain was mainly in the feet (Figure 1D). This led us to consider that linezolid caused clinical symptoms similar to diabetic peripheral neuropathy (DPN) (Liu J. et al., 2022; Fang, Wang, Song, Wang, & Zhang, 2022).

### Effects of linezolid on rats

**FIGURE 2:**
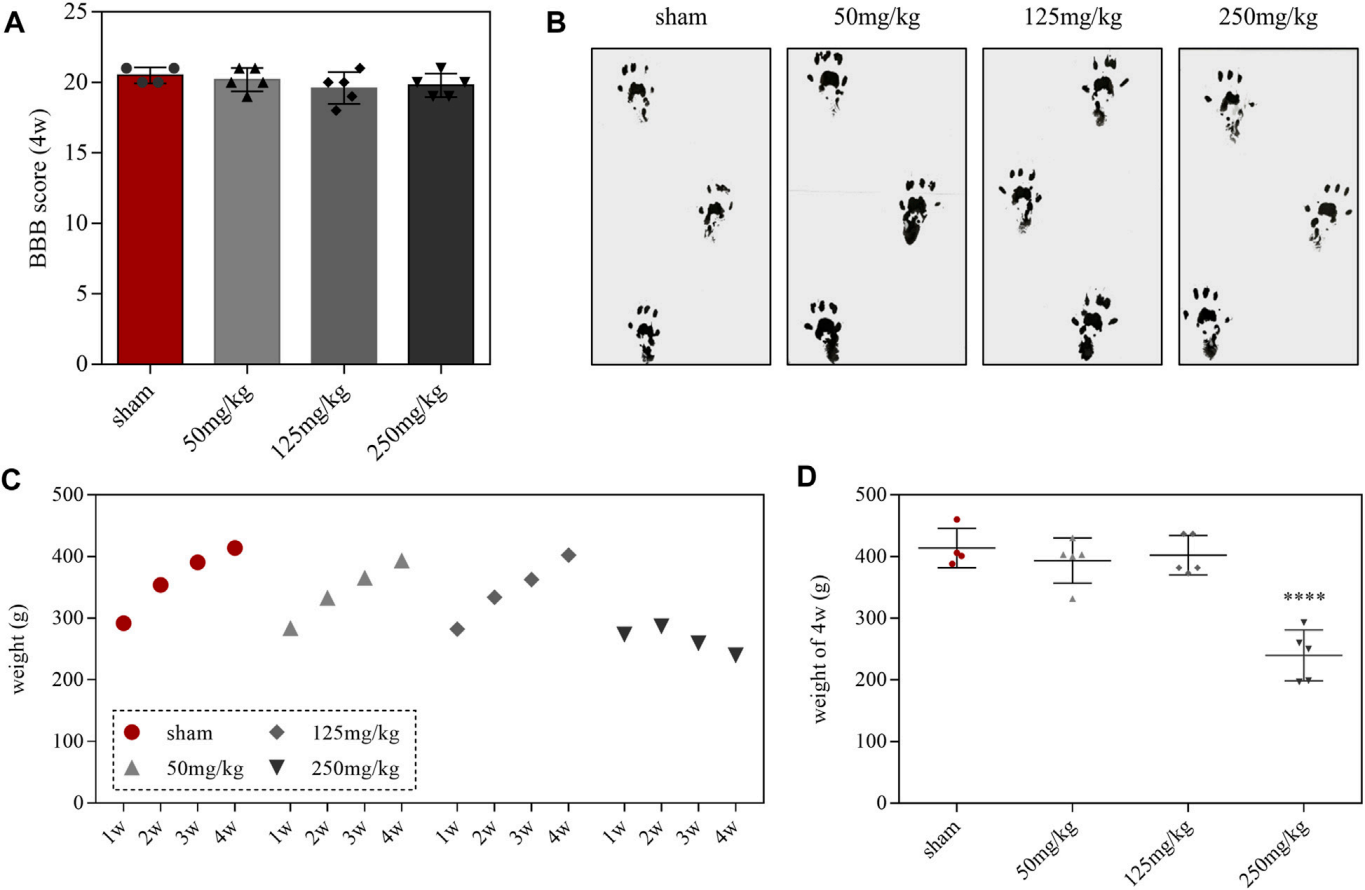
Effects of linezolid on behavior and body weight in rats. **(A,B)** Body weight records of rats in each group. **(C)** BBB scores in each group. **(D)** Walking track prints in each group at 4 weeks. Linezolid-induced neuron and myelin sheath loss.

Behavioral tests were performed after administration of linezolid for 4 weeks. BBB scores and walking track analysis showed that no abnormal motor function was observed in each group (Figures 2A,B). During the weekly monitoring of rats’ body weight, we found that the rats in the 250-mg/kg dose group exhibited moderate growth during the first 2 weeks of feeding (Figure 2C). However, compared with the sham group, the 250-mg/kg doses group presented with a significant difference in body weight on the fourth week (Figure 2D). Moreover, we also fortuitously observed that the 250-mg/kg dose group showed decreased activity and depression after 4 weeks of continuous administration.

**FIGURE 3:**
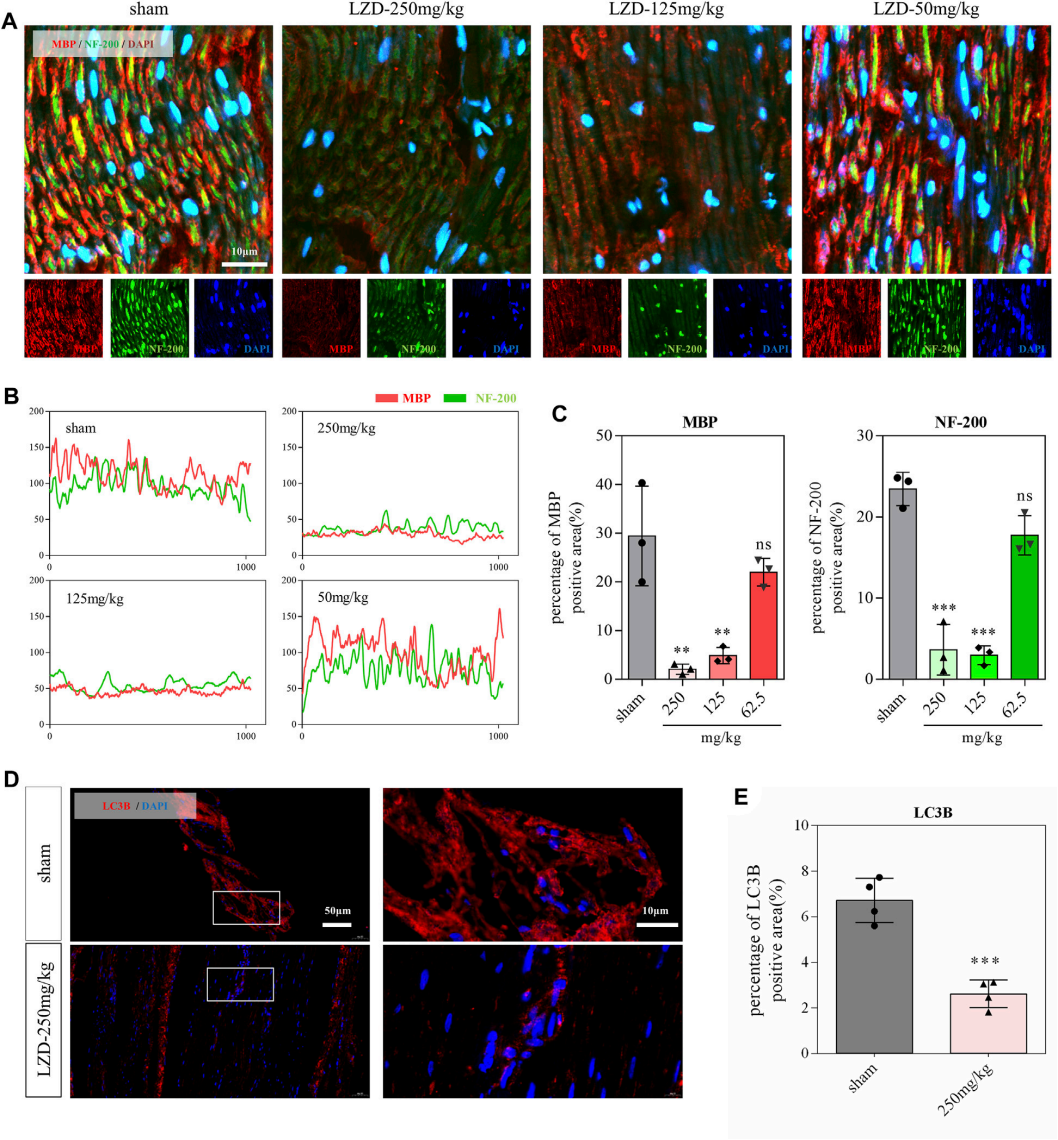
Effects of linezolid on the sciatic nerve in rats. **(A–C)** Representative images and quantitative analysis of sciatic nerve sections stained with anti-MBP antibody (red) and anti-NF-200 antibody (green) in rats (scale bar = 10 μm). **(D,E)** Immunofluorescence images and analysis of sciatic nerve in rats. Red represents anti-LC3B antibody (scale bar = 50 and 10 μm). **p < 0.01, ***p < 0.001 vs. the sham group.

The damage to neurons and myelin sheaths is a fatal blow to the nervous system (Swire et al., 2021; Liu Q. et al., 2022). In the current study, we found that nerve fibers and myelin sheaths exhibited a clear sciatic nerve structure in rats from the sham group. They displayed myelin sheath tightly wrapped laterally around the nerve fibers (Figure 3A). In the linezolid-treated group, nerve fibers and myelin sheaths were arranged sparsely, and some degree of loss in neurons and myelin sheaths was observed, which was more severe in the 250-mg/kg dose group (Figures 3A–C). Our previous studies have shown that appropriate autophagy has a neuroprotective effect and promotes peripheral nerve regeneration (Wu et al., 2019; Li et al., 2020). Through immunofluorescence staining, we observed a significant decrease in autophagic structural protein (LC3B) expression in the sciatic nerve after linezolid administration (Figures 3D,E), suggesting that linezolid contributes to the blockage of autophagy flux.

### Linezolid inhibits the proliferation of SCs

**FIGURE 4:**
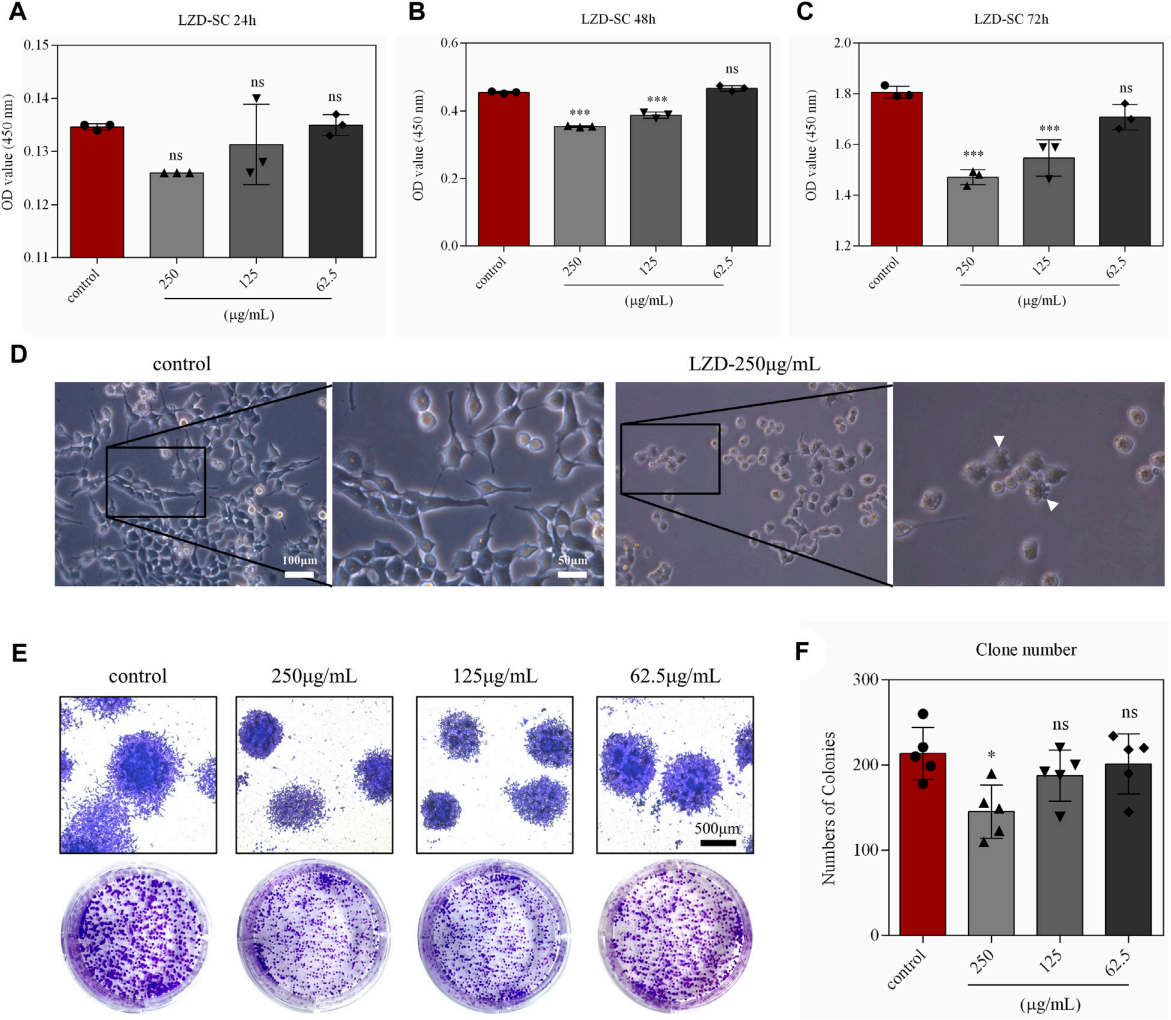
*In-vitro* simulation of linezolid-induced damage to SCs. **(A–C)** The effect of different concentrations of linezolid on SCs proliferation. **(D)** Morphology of SCs under a light microscope (scale bar = 100 and 50 μm). **(E,F)** Effects of different concentrations of linezolid on the colony-forming ability of SCs (scale bar = 500 μm). *p < 0.05, ***p < 0.001 vs. the sham group.

To better elucidate the significance of autophagy in linezolid-induced peripheral neuropathy, SCs were selected for subsequent experimental studies. Different concentrations of linezolid (62.6, 125, and 250 μg/ml) were exposed to SCs across different durations (24, 48, and 72 h) (Figures 4A–C). The results of CCK-8 assay showed that linezolid did not inhibit the growth of SCs at a concentration of 62.5 μg/ml. At the concentration of 250 μg/ml, linezolid could significantly inhibit the growth of SCs when the incubation time was 48 and 72 h, and the inhibition effect of 72 h-incubation is stronger than 48 h-incubation. After 72 h-incubation, the pseudopodia of SCs were shortened and the shape of cells was round under the light microscope (Figure 4D). The results of monoclonal experiments also confirmed SCs were significantly reduced in proliferative capacity at dose 250 μg/ml (Figures 4E,F). Given that 250 μg/ml of linezolid overtly induced toxicity in SCs at 72 h, this same concentration was used in *in-vitro* experiments.

### Linezolid inhibits the autophagy of SCs

**FIGURE 5:**
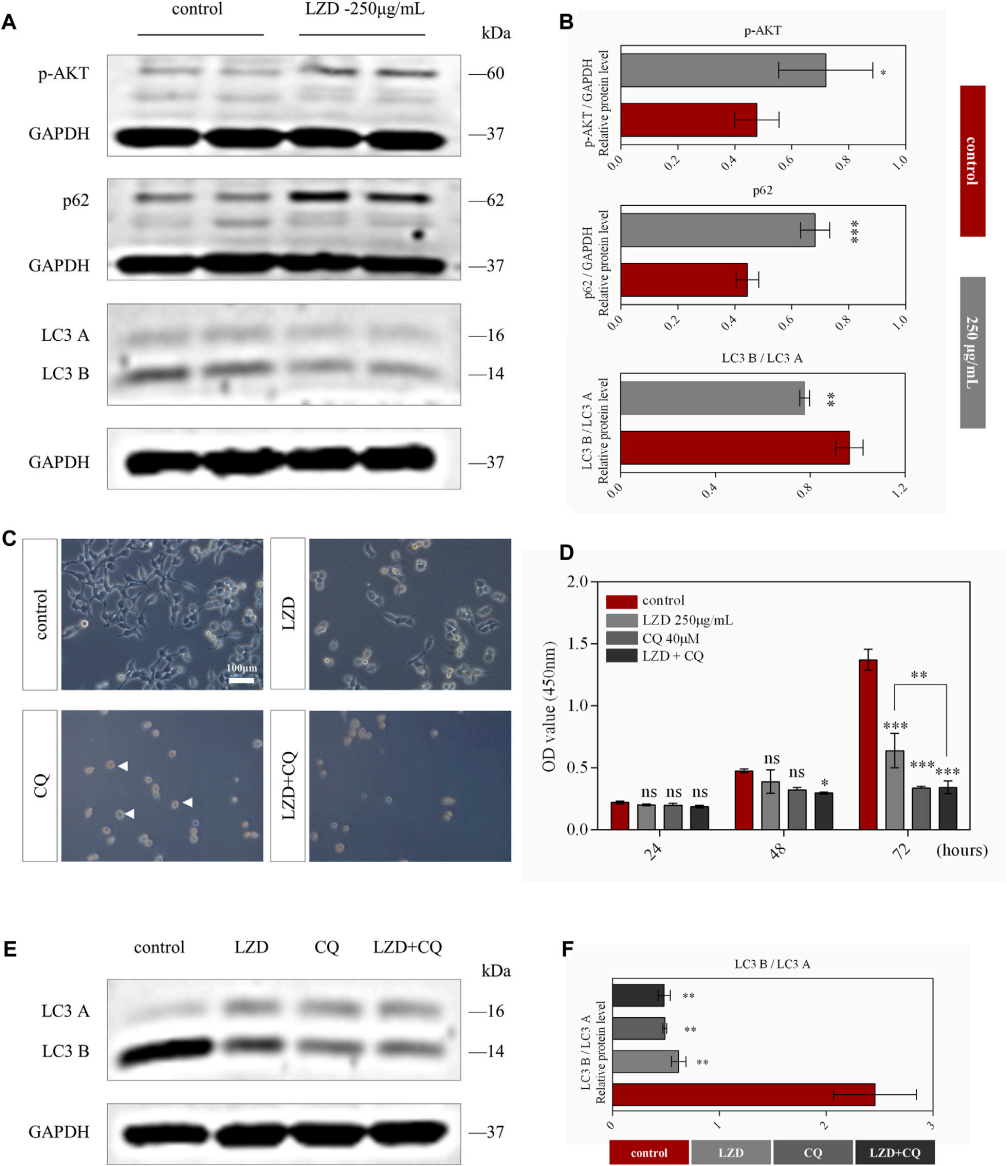
Autophagic flux was inhibited by linezolid. **(A)** Protein expression of p-AKT, p62, and LC3A/B in SCs from the linezolid-treated group. **(B)** Intensities of p-AKT, p62, and LC3A/B were normalized to GADPH. **(C)** Morphological characteristics of SCs after exposure to CQ (40 μM) and linezolid for 72 h (scale bar = 100 μm). **(D)** The proliferation of SCs in different groups was detected using the CCK8 assay. **(E)** Expression of protein LC3A/B in SCs after adding CQ and linezolid for 72 h. **(F)** Intensities of LC3A/B normalized to GADPH. *p < 0.05, **p < 0.01, ***p < 0.001 vs. the sham group.

SCs autophagy plays an essential role in peripheral neuropathy (Belgrad, De Pace, & Fields, 2020); therefore, we detected the autophagy function in our *in-vitro* model. After administering different concentrations of linezolid, we found p-AKT protein expression levels to be associated with linezolid in network pharmacology. The expression levels of p-AKT were significantly upregulated after administration, whereas the autophagy pathway of SCs was inhibited (Figures 5A,B). To further elucidate its mechanism of action, we added CQ for verification. We found that after the addition of CQ, the SCs shape changed from spindle to spherical (Figure 5C). Notably, our CCK8 assay findings further revealed that when linezolid and CQ act on SCs simultaneously, their proliferation ability is more strongly inhibited (Figure 5D). This indicated that the survival of SCs was associated with the level of autophagy and that linezolid inhibited the proliferation of SCs by inhibiting the autophagy pathway. Finally, we added linezolid and CQ at the same time and detected changes in the expression level of autophagy marker protein LC3A/B. We found that both linezolid and CQ could reduce LC3A/B expression, with the simultaneous use of linezolid and CQ promoting a more significant inhibition of LC3 A/B (Figures 5E,F).

## Discussion

A growing number of studies have focused on balancing the efficacy and toxicity of MDR-TB, but no breakthroughs have been made (Bigelow, Tasneen, Chang, Dooley, & Nuermberger, 2020; Yun et al., 2022). We collected primary data from 12 patients with MDR-TB, including gender, age, dose, duration of treatment, presence of peripheral nerve discomfort, self-assessment of pain, etc. Based on the clinical data collected herein, we found that 10 patients showed symptoms of peripheral nerve discomfort. Previous studies demonstrated that nearly half of the patients with MDR-TB had confirmed peripheral neuropathy, among which almost 78% of the cases were irreversible despite linezolid withdrawal (Falagas, Siempos, Papagelopoulos, & Vardakas, 2007; Jaspard et al., 2020). Therefore, it is necessary to elucidate the mechanism of linezolid-induced peripheral neurotoxicity and explore effective treatment.

In the current study, daily administration of linezolid in rats for 4 weeks resulted in damage to neurons and myelin sheaths in sciatic nerve tissue but did not affect behavior in rats. Mechanistic studies have found that linezolid blocks autophagy flux in rat sciatic nerve tissue and SCs, resulting in myelin sheath loss or growth inhibition. Therefore, maintaining autophagy levels may be a potential strategy to prevent or treat linezolid-induced peripheral neuropathy.

Linezolid is a synthetic oxazolidinone antibiotic used to treat severe infections caused by Gram-positive cocci (Bender et al., 2018). It acts on the 50 S subunit to inhibit protein synthesis. The existing literature lacks basic research on linezolid. Recent studies have indicated that linezolid is associated with autophagy (Abad et al., 2019; Tasneen et al., 2021). In our study, LC3B expression was significantly decreased in the linezolid-treated group. This suggests that autophagy plays a significant role in linezolid-induced peripheral neuropathy.

SCs account for 70%–80% of the components of the peripheral nervous system (PNS) (Chen, Piao, & Bonaldo, 2015; Sardella-Silva, Mietto, & Ribeiro-Resende, 2021) and spiral around the axon to form a tight myelin membrane (Della-Flora Nunes et al., 2021; Zotter et al., 2022). When peripheral nerve injury occurs, enhanced autophagy of SCs can maintain microtubule stability and promote peripheral nerve regeneration and functional recovery in the adult nervous system (Bankston et al., 2019; Li et al., 2020; Reed et al., 2020; Hu et al., 2022). Our results suggest that the proliferation of SCs is significantly inhibited when exposed to linezolid solution, along with AKT activation and inhibition of autophagy flux. Another critical feature of SCs is their ability to produce extracellular matrix and various neurotrophic factors that support the survival of damaged neurons and promote axonal regeneration, including nerve growth factor (NGF), brain-derived neurotrophic factor (BDNF), and neurotrophic factor 3 (NT-3), as well as the expression of various cell adhesion molecules on its surface (Sarhane et al., 2019; Wang et al., 2022). Therefore, we hypothesized that linezolid inhibited the survival and autophagy flux of SCs, indirectly leading to neuron cell damage. Moreover, the upregulated expression of P62 and down-regulated expression of LC3B indicated that the fusion between autophagosome and lysosome was blocked. Interestingly, the results obtained when we treated SCs with CQ were consistent with those obtained when linezolid was used on SCs.

Although our current study supports the inhibitory effect of linezolid on autophagy flux, there are still deficiencies in this study. First, we proposed that long-term use of linezolid increased the risk of peripheral neuropathy in MDR-TB, but the number of patients we collected was limited. Limited data are insufficient to assess the actual incidence of peripheral neuropathy. Second, the results do not clarify whether increased autophagy flux can salvage the viability of SCs. Finally, this study did not explore the causal relationship between SCs injury and neuron injury. Therefore, it is meaningful to improve the above deficiencies, and we will gradually improve them in future research.

**FIGURE 6:**
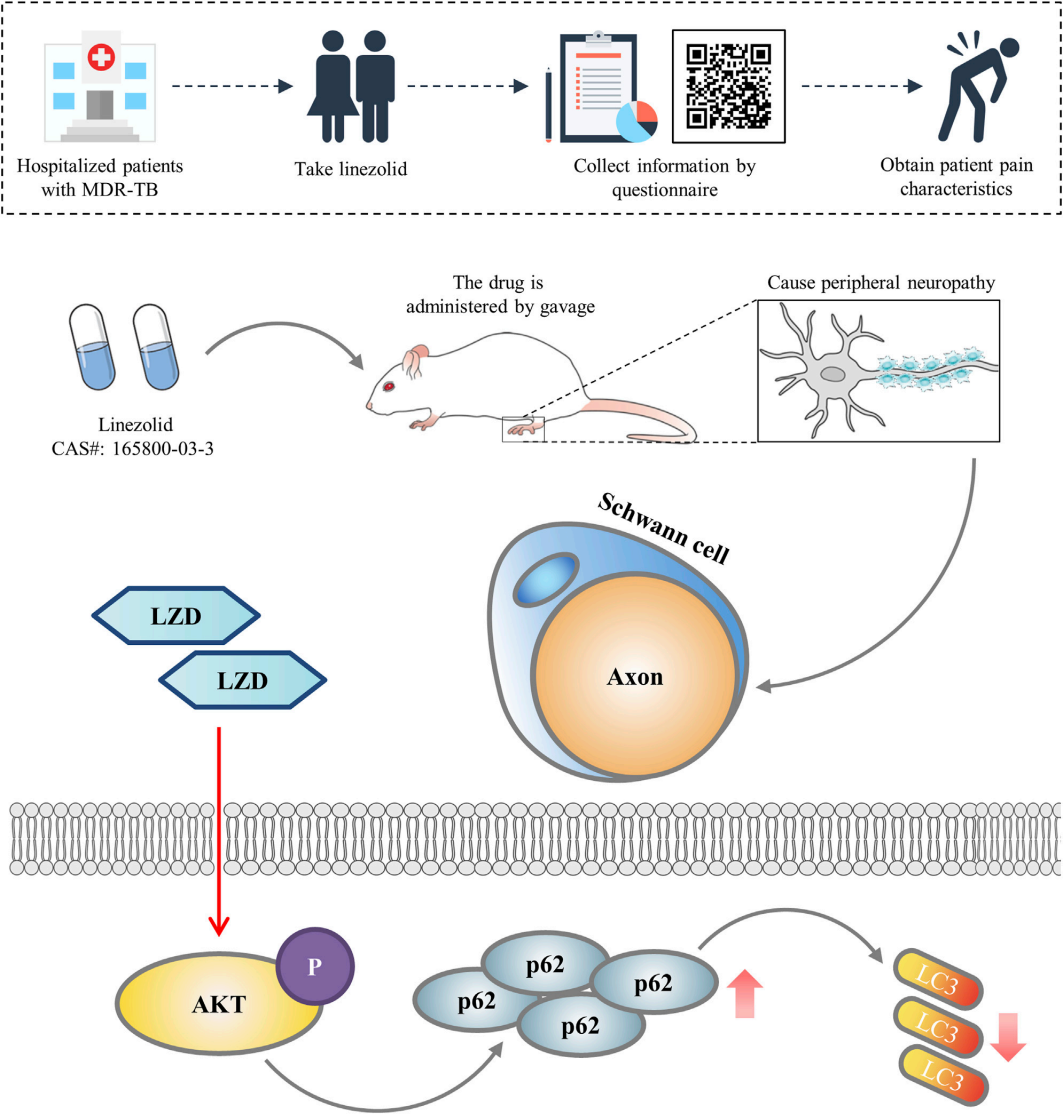
Diagram illustrating the mechanism by which linezolid induces peripheral neuropathy. We concluded that linezolid-induced peripheral neuropathy is associated with reduced autophagy flux in SCs.

In conclusion, our current study identifies the peripheral neurotoxicity of linezolid and verifies that linezolid is achieved by blocking autophagy flux (Figure 6). This may be a potential strategy to intervene in linezolid-induced peripheral neuropathy.

## Data Availability

The original contributions presented in the study are included in the article/supplementary material, further inquiries can be directed to the corresponding authors.
